# Current perspectives on the psychological stress-immune axis in asthma: Pathophysiology, interventions, and significance for patient care^☆^

**DOI:** 10.1016/j.waojou.2026.101453

**Published:** 2026-08-26

**Authors:** KaKa L. Adams, Gailen D. Marshall

**Affiliations:** Division of Clinical Immunology, Department of Medicine, The University of Mississippi Medical Center, Jackson, MS, USA

**Keywords:** Asthma, Stress, Anxiety, Depression, Psychoneuroimmunology, Immune dysfunction, Quality of life

## Abstract

Psychological stress and psychiatric comorbidities are highly prevalent in patients with asthma, with evidence supporting bidirectional links. Excessive psychological stress is associated with immune dysregulation, hypothalamic-pituitary-adrenal (HPA) axis dysfunction, and neuroimmune crosstalk that increases Type 2 inflammation associated with poorly controlled asthma. Anxiety and depression worsen asthma control and quality of life through complex and bidirectional mechanisms. Both cognitive-behavioral therapy (CBT) and mindfulness-based stress reduction (MBSR) can help reduce distress and increase quality of life for people with asthma. However, their effects on lung function and exacerbation rates remain inconsistent.

Integrating interventions that address stress and mental health factors into asthma care is essential for optimal care. Distinguishing formal psychiatric illness from stress-related emotional distress allows for specific interventions to be utilized. Embedding validated screening tools and behavioral health into routine clinical practice can address the value of managing psychosocial burden while complementing pharmacologic therapy.

## Introduction

With an estimated global prevalence of more than 250 million individuals, asthma is a chronic inflammatory disease of the airways that produces significant morbidity and persistent mortality.^1^^,^^2^ Asthma burden varies across regions and socioeconomic contexts, reflecting interactions among environmental exposures, access to care, and individual vulnerability. People with asthma have a higher prevalence of anxiety and depressive disorders than comparable individuals without asthma.^3^ The scientific discipline of psychoneuroimmunology (PNI) has provided a framework for understanding how psychological processes through mechanisms including neuroendocrine pathways such as the hypophyseal-pituitary-adrenal axis can influence immune function,^4^ a concept that extends to chronic inflammatory diseases such as asthma.

Historically, researchers and clinicians have viewed excessive psychological stress as primarily immunosuppressive, believing it weakens host defenses to increase susceptibility to infection. Living with asthma can lead to excessive emotional strain, producing a cycle in which stress and symptoms exacerbate each other. Ongoing studies indicate that persistent stress is associated with immune and neuroendocrine changes that may contribute to poor asthma control.^5^ Stress and anxiety are closely associated with more frequent exacerbations, higher healthcare utilization, and lower quality of life.^6^^,^^7^

In this review, psychological stress refers to the cognitive and emotional response that occurs when environmental, social, or internal personal demands exceed an individual's perceived ability to cope.^8^ It includes responses to chronic psychosocial stressors, adverse life experiences, and persistent emotional distress.^9^ Although psychological stress may contribute to the development of psychiatric disorders such as major depressive disorder and generalized anxiety disorder, it remains distinct from these conditions, which are diagnosed using established clinical criteria.^10^ One well-recognized form of chronic psychological stress is exposure to adverse childhood experiences (ACEs), including childhood abuse, neglect, household dysfunction, and other traumatic events.^11^ ACE exposure has consistently been associated with increased asthma prevalence, poorer asthma outcomes, and long-term alterations in neuroimmune function, illustrating the lasting impact of early-life stress on asthma across the lifespan.^12^^,^^13^

This article is not intended as a traditional narrative review. Rather, it presents a conceptual framework for understanding the psychological stress–immune axis in asthma by integrating current knowledge of underlying biologic mechanisms with potential clinical applications. The review is organized around how excessive psychological stress influences asthma through interconnected neuroendocrine, autonomic, and immune pathways, contributing to disease activity, treatment response, and patient outcomes. Using evidence from clinical, immunological, and neurobiological studies, we distinguish psychological stress exposure from formal psychiatric disease, review available interventional data, and propose an integrated approach to guide future research and asthma care.

Relevant literature was identified through PubMed using combinations of the terms asthma, psychological stress, psychoneuroimmunology, anxiety, depression, neuroimmune mechanisms, cognitive behavioral therapy, and mindfulness. Additional articles were identified through review of the reference lists of relevant publications. Studies were selected based on their relevance to the biologic mechanisms, clinical evidence, and clinical significance of the psychological stress–immune axis in asthma.

## Pathophysiologic mechanisms

**Fig. 1:**
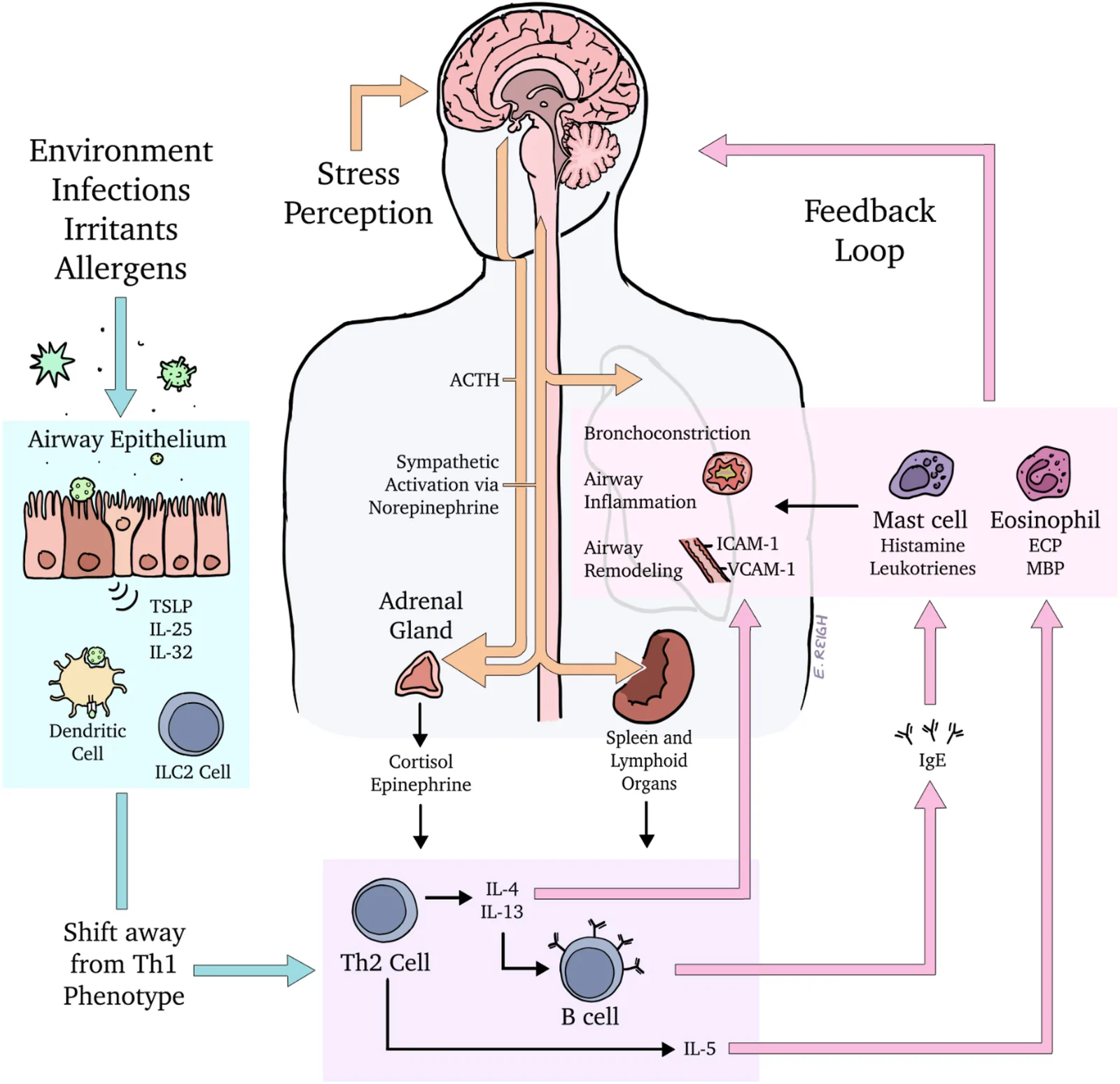
**Proposed framework linking psychological stress and asthma**. Psychological stress activates interconnected neuroendocrine and autonomic pathways that alter hypothalamic-pituitary-adrenal (HPA) axis function and sympathetic activity. These responses promote epithelial alarmin release (IL-33, IL-25, and thymic stromal lymphopoietin [TSLP]), activation of Type 2 inflammatory pathways, and downstream airway inflammation, bronchoconstriction, and airway remodeling. This proposed framework illustrates the biologic mechanisms by which psychological stress may influence asthma pathophysiology and highlights potential points at which behavioral and pharmacologic interventions may modify disease outcomes

Psychological stress can influence asthma through several interacting processes. Disruption of the hypothalamic-pituitary-adrenal (HPA) axis has been associated with altered cortisol signaling, which may limit endogenous anti-inflammatory regulation.^14^ Excessive stress is also linked with shifts in autonomic balance toward higher sympathetic activity and lower vagal tone, changes that may increase airway reactivity.^15^ Experimental and translational studies suggest that stress can promote epithelial release of alarmins such as IL-33, IL-25, and thymic stromal lymphopoietin (TSLP), mast cell activation, and downstream Type 2 (eosinophilic) inflammatory signaling, processes implicated in asthma-associated airway hyperresponsiveness.^16^ In animal models, chronic stress activates microglia and amygdala IL-1 signaling, linking peripheral airway inflammation to central neuroinflammatory changes associated with anxiety-like behaviors.^17^^,^^18^ Human neuroimaging studies further suggest that stress-related amygdala activity is associated with increased airway IL-1 signaling and may contribute to neuroimmune interactions relevant to asthma.^19^ Together, these findings support a biologic link between excessive psychosocial stress and increased asthma severity (Fig. 1).

Emerging evidence suggests that chronic psychological stress may also be associated with changes in gene regulation through epigenetic mechanisms including DNA methylation and histone acetylation that can affect genes involved in cytokine signaling and glucocorticoid receptor function. These alterations have been linked to heightened airway inflammation and reduced glucocorticoid responsiveness, particularly in children exposed to early-life adversity. Social and environmental stressors, such as financial hardship or limited access to medical resources, may further shape these epigenetic patterns.^20^^,^^21^

Chronic activation of stress-related pathways may contribute to a self-reinforcing cycle in which neuroendocrine, autonomic, and immune signals interact to allow or even promote sustained airway inflammation. Reduced vagal tone and elevated catecholamines have been linked to dendritic cell activation and immune polarization toward Type 2 responses,^16^ while impaired HPA axis feedback may diminish cortisol-mediated restraint of inflammation.^5^^,^^14^^,^^15^ Over time, these neuroendocrine–immune interactions have been associated with features of decreased corticosteroid sensitivity and airway remodeling, linking psychosocial stress with persistent disease activity and suboptimal treatment response.^5^^,^^14^^,^^15^^,^^20^ Recognizing these interacting pathways provides substantial mechanistic rationale for development and implementation of integrative approaches that address both immune and psychosocial contributors to asthma burden. This can promote the goal of optimizing response to standard controller therapy and biologic treatment in selected patients.

## Distinguishing psychiatric disease from psychosocial-related distress

Distinguishing psychological stress exposure from formal psychiatric disorders, such as major depressive disorder and generalized anxiety disorder, is important. Psychological stress is an exposure, whereas psychiatric disorders are clinical diagnoses defined by established diagnostic criteria. Although psychological stress may contribute to the development or worsening of psychiatric illness, it may also independently influence asthma through neuroendocrine, autonomic, and immune mechanisms.^13^ Both psychological stress and psychiatric disorders can worsen asthma outcomes through overlapping biological and behavioral pathways, but they present different clinical challenges. Psychiatric disorders often require pharmacologic treatment and structured psychotherapy, whereas stress-related distress may respond to resilience training, coping strategies, or integrative therapies.^22^^,^^23^ Recognizing this distinction can help sharpen research questions and guide more individualized patient care.

## Interventions and outcomes

Psychological interventions may improve mental health and daily functioning in patients with asthma, although consistent evidence for sustained clinical benefit remains limited. Studies suggest that mindfulness-based stress reduction and cognitive-behavioral therapy may help reduce perceived stress while improving asthma-related quality of life.^24^, ^25^, ^26^ Emerging evidence suggests that digitally delivered or telehealth-based mindfulness and cognitive-behavioral interventions may improve access to behavioral support for individuals with asthma, although additional studies are needed to establish their long-term clinical effectiveness.^25^ Defined breathing practices may further support symptom awareness and stress regulation.^27^ Structured techniques, including diaphragmatic breathing, pursed-lip breathing, and yoga-based pranayama, have shown benefit in lowering perceived stress and improving symptom control in some studies, although consistent effects on inflammatory or physiologic markers in asthma have not been demonstrated.^27^^,^^28^

Because psychological stress is a potentially modifiable exposure, interventions should extend beyond treatment of psychiatric disease alone. Strategies that reduce ongoing psychosocial stress, strengthen resilience, and improve coping skills may help lessen the physiologic effects of chronic stress. Mindfulness-based stress reduction, cognitive behavioral therapy, structured breathing exercises, physical activity, and stress management and resilience-building approaches have shown promise in improving perceived stress and quality of life, although evidence for consistent improvements in objective asthma outcomes remains limited.^24^, ^25^, ^27^^,^^29^ Addressing social determinants of health and chronic psychosocial adversity may also help reduce stress exposure, although additional research is needed to determine whether these approaches improve asthma specific outcomes.^30^

Psychological therapies should therefore complement, rather than replace, standard pharmacologic treatments such as inhaled corticosteroids, beta adrenergic agents and biologics. Integrated asthma care may also include lifestyle, behavioral, and medical strategies

**Fig. 2:**
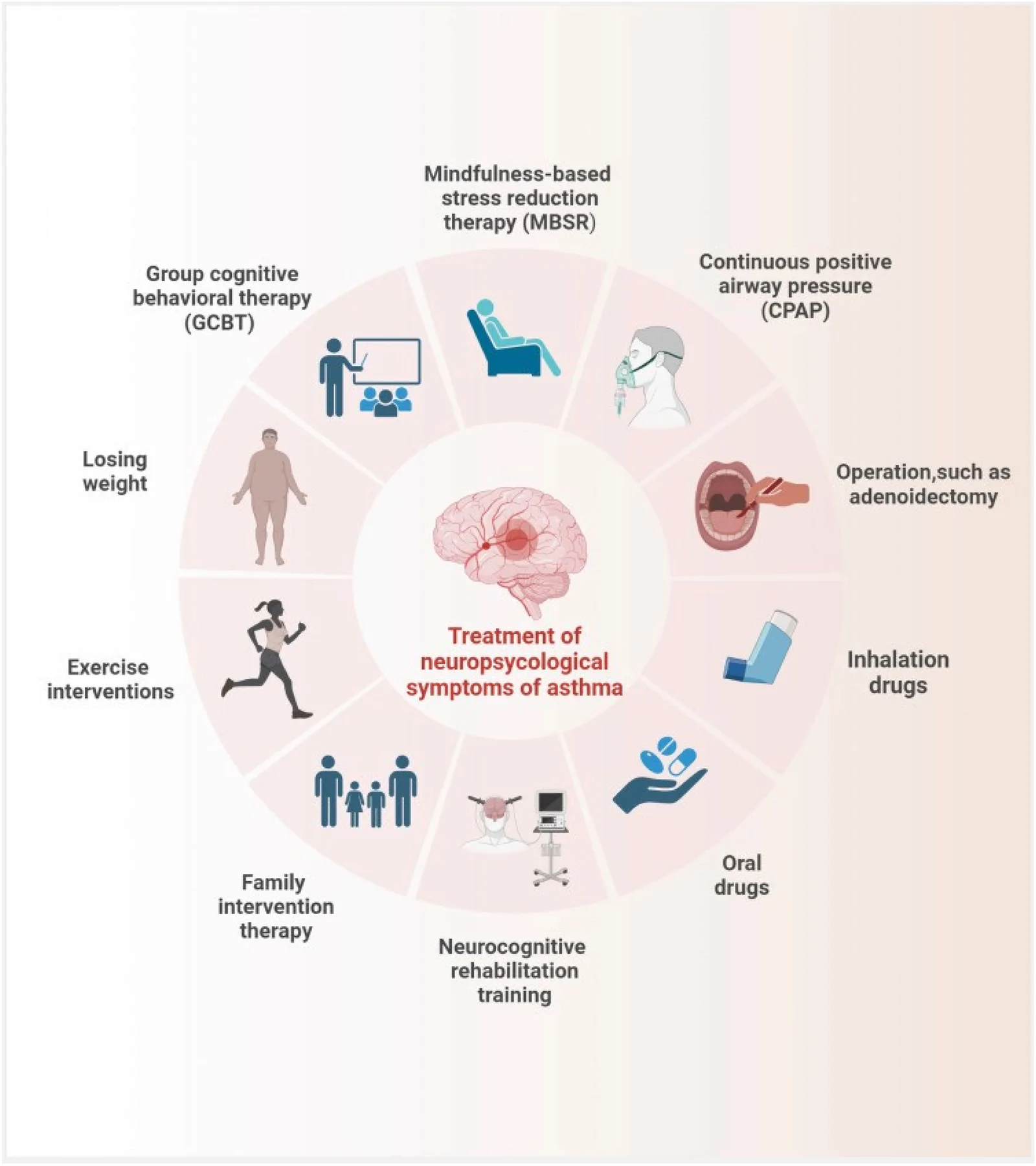
**Potential intervention strategies targeting psychological stress and asthma outcomes**. This framework summarizes behavioral, lifestyle, and medical interventions that may reduce the impact of psychological stress on asthma. Psychological interventions, including mindfulness-based stress reduction, cognitive behavioral therapy, family based interventions, exercise, and resilience building strategies, may help reduce stress related physiologic responses. Standard asthma therapies and management of relevant comorbidities remain the foundation of asthma care and should be used alongside behavioral and psychosocial interventions when appropriate

Such as exercise programs, weight management, family-based interventions, treatment of sleep-disordered breathing, and pharmacologic therapy, highlighting the range of approaches that can influence both asthma control and psychological well-being (Fig. 2). Pharmacologic treatment of anxiety or depression, for instance with antidepressants, may reduce underlying psychosocial burden, but it is unlikely on its own to address stress-related neuroimmune pathways that are playing a role in poor asthma control.

## Bidirectional relationship between stress and asthma

Psychological stress may worsen asthma through neuroendocrine, autonomic, and immune dysregulation,^13^ whereas poorly controlled asthma may increase psychological stress through symptom burden, activity limitation, sleep disruption, emergency health care utilization, and uncertainty regarding disease control.^31^, ^32^, ^33^ Together, these processes may create a reinforcing cycle that contributes to worsening asthma outcomes and reduced quality of life. Understanding this bidirectional relationship supports an integrated approach to care that addresses both asthma management and psychosocial well-being.

## Broader context gathered from other diseases

Emotional stress and psychological hardship are linked with a range of medical conditions in addition to asthma. Depression has been linked to poorer recovery following myocardial infarction.^34^ Stress is known to intensify clinical symptoms and adverse biomarkers in inflammatory conditions such as lupus, rheumatoid arthritis, and inflammatory bowel disease.^35^ In dermatologic illnesses such as psoriasis and atopic dermatitis, stress and depression frequently coexist, illustrating the influence of neuroimmune pathways.^36^ Together, these observations suggest that psychoneuroimmunologic mechanisms may represent a common pathway across several chronic inflammatory diseases.

## Evidence gaps and limitations

While many studies show a link between excessive psychological stress and poor asthma outcomes, the overall evidence has important limitations which require consideration. A substantial portion of the existing literature consists of observational studies that depend on patient-reported assessments of psychosocial stress or adversity, limiting the ability to fully account for bias and unmeasured, potentially confounding variables.^12^ Psychosocial and socioeconomic stressors frequently occur alongside other environmental exposures including pollens, animal dander, mold spores, dust mite proteins, tobacco smoke and air pollution, as well as social determinants of health including limited access to care and medication costs, any of which may themselves contribute to stress levels in patients and their families. This can contribute to difficulty in separating the various stress-related and environmental factors influencing asthma control in individual patients. Prior studies show that lower socioeconomic status is associated with poorer asthma control, higher emergency care utilization, and lower self-efficacy in asthma management.^37^^,^^38^ Results from interventional studies have been varied. Trials of cognitive-behavioral therapy and mindfulness-based approaches tend to show the most consistent benefits in patient-reported outcomes, such as emotional distress and asthma-related quality of life, while effects on objective measures, including lung function and biomarkers, have been less consistent across studies.^24^^,^^29^ Many studies are small in sample size, include diverse asthma populations, and differ in type and duration of specific interventions. Accordingly, there is wide variation in outcome measures, making comparisons harder and limiting direct applicability of the findings.^29^^,^^39^ There is growing evidence for neuroimmune pathways in asthma, but much of the mechanistic data come from experimental models or limited, highly controlled human studies, making it difficult to draw conclusions about causality.^40^ Overall, the existing evidence supports the idea that excessive psychological stress meaningfully influences asthma burden. This highlights the need for more studies with stronger causal designs and more standardized outcome measures.^41^

## Future directions

To help close these gaps, future research efforts should focus on designs that go beyond cross-sectional associations and better support causal inference. Prospective longitudinal studies that follow psychosocial stress and asthma outcomes over time can help clarify temporal relationships between excessive stress exposure and disease activity. Applying standardized, validated tools with clear thresholds will be important for distinguishing stress-related emotional distress from formal psychiatric disease, recognizing that both can influence asthma outcomes. Studies should consistently include both patient-reported and objective asthma outcomes, such as exacerbation frequency, systemic corticosteroid use, lung function, and health care utilization.

Intervention trials should focus on outcomes that matter in everyday clinical practice. Real-world studies embedded in routine asthma care could evaluate cognitive-behavioral therapy, mindfulness-based programs, and structured breathing interventions, using stepped-care approaches.^42^, ^43^ Such models may include screening-based referral to mental health professionals, psychotherapy, and/or adjunctive pharmacologic treatment when clinically appropriate. Consistently tracking shared outcomes, including quality of life, symptom control, asthma exacerbations, medication use, and lung function, would help identify which strategies deliver the greatest clinical benefit. Adequate follow-up is also needed to capture seasonal variation and delayed effects, given the heterogeneity and short follow-up periods noted in existing trials.^39^

Future efforts should also focus on developing evidence based consensus recommendations to guide implementation of psychosocial screening and behavioral health integration into routine asthma care.^44^ The experience of the cystic fibrosis community provides a useful model, where international consensus guidelines have standardized screening and management of depression and anxiety in patients and caregivers.^45^ Similar multidisciplinary recommendations for asthma could facilitate broader adoption of psychological assessment, behavioral health integration, and coordinated multidisciplinary care in routine clinical practice.

Additional work is needed to better understand how psychosocial stress affects immune and neuroendocrine pathways by linking clinical observations with biological data.^46^, ^47^ Key areas of interest include stress-induced changes in hypothalamic-pituitary-adrenal axis activity across diverse patient populations and asthma phenotypes, autonomic nervous system markers, epithelial alarmins, Type 2 inflammation profiles, and organ-based glucocorticoid signaling.^5^^,^^14^^,^^16^^,^^20^ Integrating biologic markers with epigenetic profiling and, when feasible, neuroimaging may help clarify mechanisms linking psychological stress with immune dysregulation in asthma. Future studies examining functional brain networks together with immune biomarkers and clinical outcomes may further define how alterations in brain function influence airway inflammation and asthma severity while identifying novel targets for behavioral and pharmacologic interventions.^15^^,^^19^^,^^48^ Subsequent research should continue to explore whether stress-related biological changes have consistent effects on disease patterns and should prioritize addressing the role of health disparity. Prior work shows that social and structural determinants, including transportation challenges, medication costs, fragmented care, and limited access to behavioral health services, contribute to psychological distress and poorer asthma outcomes.^30^ Future studies should examine whether interventions that reduce these barriers improve the effectiveness of psychological and behavioral approaches. Research conducted in communities with a high asthma burden should also assess the feasibility, acceptability, and expandability of integrated care strategies to reduce persistent disparities in asthma outcomes.^30^

## Implementation in clinical practice

Applying psychoneuroimmunology in asthma care requires practical screening tools and a structured approach to patient evaluation. Screening may be considered at diagnosis, during step-up or step-down therapy, after exacerbations, and as part of routine follow-up. Commonly used tools include the PHQ-9 for depression, the GAD-7 for anxiety, and the Hospital Anxiety and Depression Scale (HADS), which was developed for use in medical settings to minimize overlap between psychiatric and somatic symptoms.^49^ The Asthma Control Test (ACT) is a validated measure of asthma control and is widely used in both clinical practice and research.^50^ Measures of asthma-related quality of life and psychosocial distress may complement symptom-based assessments by helping clinicians better understand how emotional distress affects patients with asthma. Assessment of psychological stress exposure may also be appropriate in selected patients because stress exposure is distinct from psychiatric disease. Instruments such as the Adverse Childhood Experiences (ACEs) questionnaire^11^ may help identify individuals with significant chronic stress exposure alongside screening for depression and anxiety.

A stepwise approach may facilitate translation of these concepts into clinical practice. In routine clinical care, mood, anxiety, and asthma control can be assessed using commonly used tools such as the PHQ-9, GAD-7, and ACT. Screening tools should support clinical decision-making, with management guided by symptom severity, functional impact, and safety considerations. Patients with mild symptoms, including PHQ-9 or GAD-7 scores in the mild range, may benefit from education, reassurance, stress-management strategies, and reassessment at subsequent visits. By contrast, patients with moderate to severe symptoms, persistent distress despite supportive measures, functional impairment, or safety concerns, including suicidal ideation, should be referred to a behavioral health clinician for further evaluation and management. When medication is considered, pharmacologic options, such as selective serotonin reuptake inhibitors, can follow a comprehensive psychiatric evaluation and shared decision-making rather than being initiated routinely within asthma clinics. For many patients, psychotherapy, particularly cognitive behavioral therapy, is supported as an initial approach, with pharmacologic treatment reserved for those with diagnosed mood or anxiety disorders or for those who do not improve with nonpharmacologic strategies.^26^ Clinicians caring for patients with asthma play a key role in recognizing individuals who may benefit from further evaluation and supporting prompt referral, while ongoing care coordination helps ensure that mental health treatment is consistent with asthma management.^23^^,^^33^

If results fall within the moderate-to-severe range, a medical provider should refer the patient to a behavioral health professional. Treatment may involve cognitive-behavioral therapy and mindfulness-based programs to strengthen coping and self-management skills. Ongoing follow-up with repeated psychosocial and asthma assessments helps track progress over time. A recent study found that children and adolescents with asthma have higher rates of anxiety and related symptoms, underscoring the importance of routinely assessing both asthma control and mental health. Fleischer and colleagues reported that anxiety symptoms are elevated in youth with asthma, supporting the need for clinicians to assess emotional distress and consider mental health referral when indicated.^51^

Striving for equity in asthma care still remains an ongoing challenge. Limited financial resources and barriers to care can compound psychological distress and contribute to poorer asthma control. Individuals from lower-income and historically disadvantaged communities face an increased prevalence of anxiety and depression related to asthma and frequently encounter barriers to accessing behavioral health care.^30^ These inequities demonstrate the need for integrative culturally responsive care models that integrate psychosocial screening and behavioral interventions into routine asthma management.

While documenting clinical encounters, clinicians should describe associations carefully, for instance by noting that psychological distress is linked with poorer asthma control, and clarify that psychological interventions are intended to support rather than substitute for standard asthma treatment.^33^ Beyond screening and referral, behavioral health support can be incorporated directly into asthma care through collaborative care models that include behavioral health clinicians, social workers, or behavioral health coordinators in pulmonary or allergy clinics. Evidence from collaborative care approaches in chronic illness demonstrates improvements in treatment adherence, symptom burden, and quality of life, although effects on disease-specific outcomes such as exacerbations may be variable.^52^

## Conclusion

Excessive stress and psychological comorbidities exert measurable effects on asthma pathophysiology and outcomes. By altering HPA axis function, autonomic balance, and neuroimmune signaling, stress can increase inflammation and symptom burden. At the same time, asthma morbidity contributes to anxiety and depression, creating a self-sustaining cycle.

Systematic reviews suggest that cognitive-behavioral therapy can improve quality of life and mental health in individuals living with chronic medical conditions. Integrating behavioral health approaches and prioritizing equity in asthma care are, therefore, important, although evidence for consistent physiologic benefit remains limited. Clarifying how stress and immune pathways interact may help translate mind–body research into practical improvements in asthma management.

## Author contribution

All authors have access to the data and a role in writing the manuscript.

## Declaration of generative AI and AI-assisted technologies in the writing process

No GenAI or other AI-assisted technologies were used.

## Funding

Supported in part by The R. Faser Triplett Sr MD Chair of Allergy and Immunology fund.

## Conflicts of interest

No conflicts of interest.
